# Association between short-term fine particulate matter exposure and hospital-acquired pneumonia risk in critically ill post-cardiac surgery patients: a 10-year cohort study using distributed lag non-linear models

**DOI:** 10.3389/fpubh.2026.1867760

**Published:** 2026-10-22

**Authors:** Tianshuo Li, Yuanyuan Yu, Chenmin Liu, Zhiheng Liu, Qi Tan, Wang Li

**Affiliations:** 1 Intensive Care Unit (ICU), Department of Cardiac Surgery, Shandong Provincial Hospital Affiliated to Shandong First Medical University, Jinan, China; 2 Department of Medical Data, School of Public Health, Cheeloo College of Medicine, Shandong University, Jinan, China; 3 Institute for Medical Dataology, Cheeloo College of Medicine, Shandong University, Jinan, China; 4 Department of Anesthesiology, Shandong Provincial Hospital Affiliated to Shandong First Medical University, Jinan, Shandong, China

**Keywords:** air pollution, cardiac surgery, critical care, hospital-acquired pneumonia, particulate matter

## Abstract

**Background:**

Ambient air pollution (AAP) exposure is a substantial health threat to humans globally. However, whether AAP could promote hospital-acquired pneumonia (HAP) onset in critically ill patients, a vulnerable population undergoing cardiac surgery in the intensive care unit (ICU), remains elusive. This study evaluated the correlation of HAP onset with fine particulate matter (PM_2.5_).

**Methods:**

In total, this study included 6,196 patients who underwent cardiovascular surgery at the ICU of a major hospital in Shandong province, China, over 10 years, from January 1, 2014, to December 31, 2023. The daily mean levels of sulfur dioxide (SO_2_), PM_2.5_, carbon monoxide (CO), PM_10_, and ozone (O_3_) were assessed from publicly available data. The outcome was HAP, defined according to CDC/NHSN pneumonia criteria as pneumonia developing >48 h after hospital admission and not present or incubating at admission; VAP was not analyzed as a separate endpoint. The association between PM_2.5_ and HAP was estimated *via* a distributed lag non-linear model, adjusted for pollutants, time trends, and weather conditions.

**Results:**

This study analyzed 1938 (31.278%) HAP cases. The mean levels of PM_10_, O_3_, PM_2.5_, and SO_2_ were 120, 75, 59, and 25 μg/m^3^, respectively, and the mean CO level was 0.979 mg/m^3^. The association between PM_2.5_ and HAP was statistically significant at lag 1 day within the perioperative exposure window. At a PM_2.5_ concentration of 30 μg/m^3^ compared with the reference concentration of 10 μg/m^3^, 14.2% higher odds of HAP were observed (95% CI: 2.6–27%). The stratified analyses indicated stronger associations among elders aged ≥ 60 years, patients with body mass index (BMI) > 23.9 kg/m^2^ and patients with diabetes. Additional adjustment for clinical covariates, including duration of mechanical ventilation, confirmed the robustness of the observed exposure–response association.

**Conclusion:**

These findings revealed a substantial and robust link between short-term PM_2.5_ exposure and elevated HAP incidence in the vulnerable population undergoing cardiovascular surgeries.

## Introduction

Air pollution (AP) is among the greatest environmental and health concerns globally (1). In 2021, it was estimated that particulate matter (PM) AP was the primary cause of the global disease burden, accounting for 8.0% of total disability-adjusted life-years (2). AP has been shown to comprise various components, including airborne PM, nitrogen dioxide (NO_2_), ozone (O_3_), and sulfur dioxide (SO_2_) (3). Fine PM (aerodynamic diameter < 2.5 μm, PM_2.5_) is the most crucial air pollutant and is the predominant environmental risk factor for human health globally (4). Several studies have evaluated the impact of PM _2.5_ on elevated death and morbidity, emergency visits, and hospitalization for acute cardiovascular and respiratory events, such as asthma, acute heart failure, pneumonia, acute coronary syndrome, chronic obstructive pulmonary disease (COPD), and stroke (5–8). Although PM_2.5_ can affect the function of various organs, most previous studies have investigated outdoor rather than indoor exposure (even though people spend most of their time indoors) and chronic rather than short-term effects of PM. Zhang *et al.* indicated that air purifiers can improve indoor air quality, and after 7 days, the forced vital capacity marginally increased (9). Our previous study demonstrated that ambient AP (AAP) can increase PM_2.5_ levels, as it can spread indoors; therefore, the indoor PM_2.5_ content is generally higher when outdoor PM_2.5_ is increased (10). Multiple studies have evaluated the influence of indoor air quality on the performance of office workers (11, 12); however, the effect of PM_2.5_ on the pulmonary performance of people in specific locations, such as the intensive care unit (ICU), remains undetermined.

Hospital-acquired pneumonia (HAP) is the most prevalent infection in the ICU (13) and is described as pneumonia that is not in the incubation period at the time of admission and occurs > 48 h after admission (14). The HAP-related mortality is about 5–13% despite improvements in prevention and treatment (15, 16). However, whether AP can increase HAP incidence has not been comprehensively studied. The smaller the particle, the deeper it can penetrate the respiratory system, causing cytotoxicity, inflammation at the alveolar level, and possible mutagenesis (17). Therefore, AAP is a substantial risk factor for reduced pulmonary function, respiratory infections, and progression of existing pulmonary disease in patients undergoing major surgeries. A Philippine study indicated that the prevalence of hospital-acquired infections in the ICUs was 17/60 (28.3%), and that pneumonia was the most common HAP (18). Furthermore, AP exposure had an immediate and delayed effect on cardiovascular diseases and respiratory diseases, respectively (19). Previous literature has investigated only the relatively coarse phase, focusing on outdoor ambient pollution exposure and its association with clinical outcomes (19), and only a few studies have assessed the air quality in ICUs and its delayed effects on HAP. Our previous study on ICU indoor air quality showed a strong correlation between indoor and outdoor PM_2.5_ concentrations in mechanically ventilated ICUs. Furthermore, short-term exposure to PM_2.5_ was found to be a potent risk factor for Ventilator-associated pneumonia (VAP) onset in pediatric patients undergoing cardiovascular surgery (10). In contrast, the present study focused on HAP in adult postoperative cardiac surgery patients, and VAP was not analyzed as a separate endpoint. However, whether ambient pollution significantly affects older adults cardiovascular patients remains unknown.

Short-term exposure to PM_2.5_ may influence the development of HAP through both immediate and delayed biological pathways. Fine particles can penetrate deeply into the lower respiratory tract, induce oxidative stress and alveolar inflammation, impair mucociliary clearance, and alter host immune responses, which may increase susceptibility to pulmonary infection over subsequent days rather than only on the day of exposure (20–22). In addition, the exposure–response relationship between PM_2.5_ and respiratory outcomes may be non-linear, particularly in settings with relatively high and variable pollution levels (23, 24). Therefore, models based on a single lag day or a simple moving-average exposure may not adequately capture the complex exposure–lag–response pattern. The distributed lag non-linear model (DLNM) provides a flexible framework to simultaneously estimate non-linear exposure–response associations and delayed effects across multiple lag days, thereby allowing assessment of both lag-specific and cumulative risks (10, 25, 26). This approach is particularly suitable for evaluating the short-term impact of PM_2.5_ on HAP.

China is the largest developing country experiencing the most severe AP problems. Therefore, short-term PM_2.5_ exposure may contribute to an increased incidence of HAP in this vulnerable postoperative ICU population. This epidemiological study aimed to elucidate the association between short-term PM_2.5_ exposure and HAP risk among adult patients undergoing cardiac surgery. We applied a DLNM to characterize the potentially non-linear and delayed exposure–lag–response relationship between PM_2.5_ exposure and HAP onset.

## Methods

### Study population

This single-center retrospective cohort study collected the medical data of all adult patients undergoing cardiovascular surgeries at the ICU of the East Campus of Shandong Provincial Hospital Affiliated to Shandong First Medical University (Shandong, China), from January 1, 2014, to December 31, 2023. This study was authorized by the ethical committee of Shandong Provincial Hospital Affiliated to Shandong First Medical University (SZRJJ: No. 2025–372).

Patients who were long-term residents of Jinan city or areas with meteorological air quality index (AQI) within 10% of the Jinan index, and who underwent cardiovascular surgery, were included in the study. Patients who stayed in the ICU for <2 days (n = 264), had pneumonia up to 3 days before surgery (n = 31), lived in cities with >10% difference in PM_2.5_ levels compared with Jinan city (n = 529), or had missing HAP data (n = 37) were excluded. These exclusion criteria were applied to ensure an adequate observation window for HAP diagnosis, avoid misclassification of pre-existing pneumonia as postoperative HAP, and reduce exposure misclassification when assigning ambient PM_2.5_ exposure from Jinan monitoring data. In total, 6,196 individuals were included in this analysis (Figure 1).

**Figure 1:**
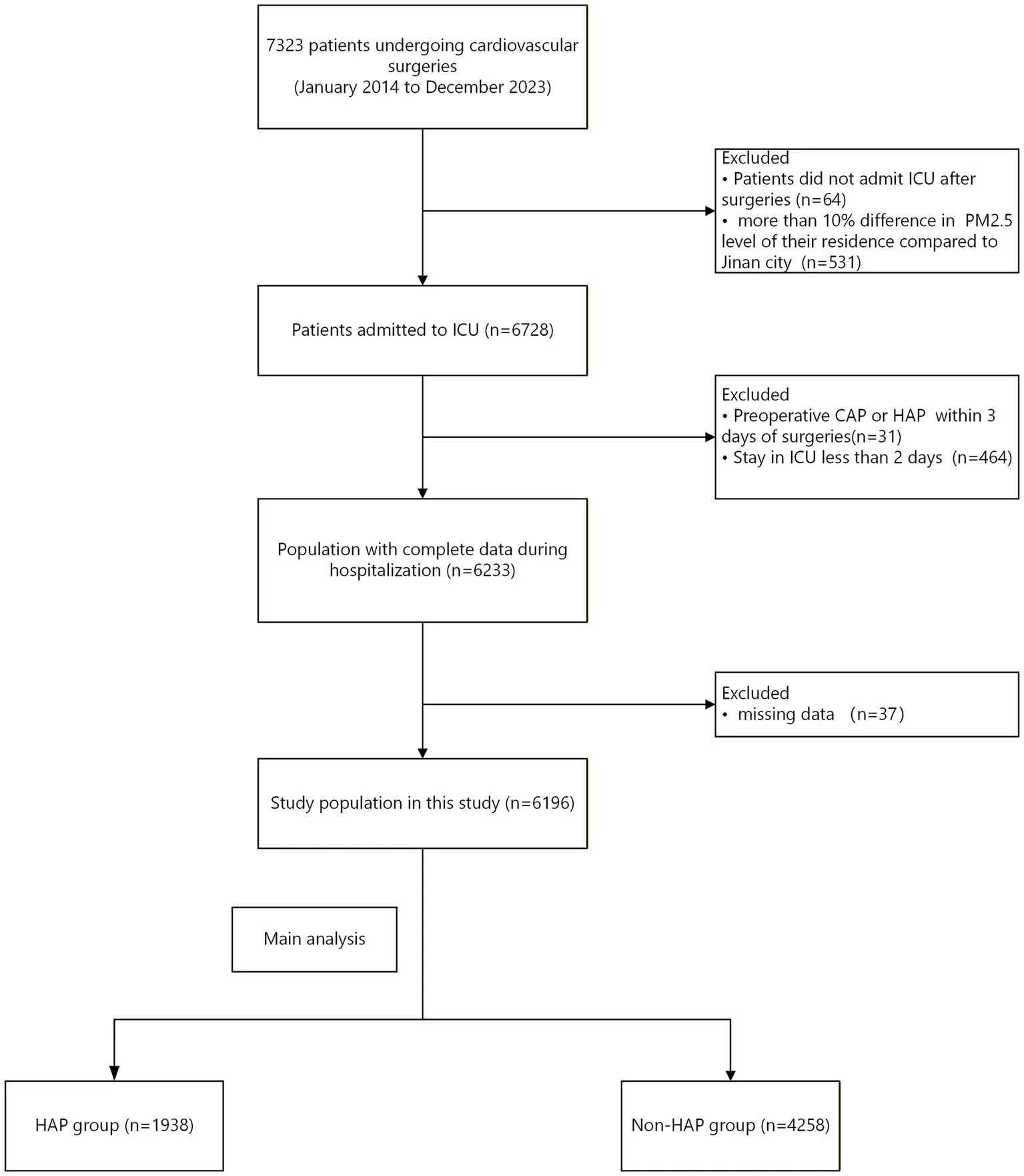
Schematic of patient selection strategy. ICU, Intensive care unit; HAP, hospital-acquired pneumonia; CAP, Community-acquired pneumonia.

### AP and meteorological conditions in Jinan

Jinan is situated at the eastern extremity of the Hanghe River basin and experiences a subtropical monsoon climate, with a population of 10.5 million. The Jinan Environment Monitoring Center provided data on the 24 h mean levels of following indices: fine particles (PM_2.5_), sulfur dioxide (SO_2_), inhalable particles (PM_10_), carbon monoxide (CO), AQI, wind force, maximum 8 h average levels of ozone (O_3_), humidity, maximum and minimum temperature (T_max_ and T_min_), and wind direction. The AQI categories included good (0–50), moderate (51–100), unhealthy for sensitive groups (101–150), unhealthy (151–200), very unhealthy (201–300), and hazardous (301–500). The Chinese government has implemented extensive quality assurance and quality control protocols at every stage. Thus, assessments of air pollutants may indicate the overall exposure of the population to urban background AP.

### Outcome

The study outcome is HAP, defined according to CDC/NHSN pneumonia criteria as pneumonia (a lower respiratory tract infection confirmed by a new pulmonary infiltrate on imaging) that develops > 48 h after hospital admission and was not present or incubating at the time of admission (27). VAP was not analyzed as a separate endpoint. HAP was diagnosed by 2 clinicians, and patients with missing or unverified data were removed from the investigation.

### Missing data

This investigation had a low percentage of missing data, and the missing values for baseline and perioperative variables are presented in Table 1. Missing values were observed for BMI, smoking status, diabetes, hypertension, infarction history, cardiopulmonary bypass, LCOS, duration of mechanical ventilation, and ICU stay. No imputation was performed, and analyses were conducted using complete cases for the variables included in each model.

**Table 1:** Clinical characteristics of patients involved in the study.

Variable	Missing, *n* (%)	Overall (6196)	HAP = 0 (4258)	HAP = 1 (1938)	*X* ^2^	*p*-value
Gender (M, proportion)					0.34	0.56
Female	0	2,411 (38.912%)	1,646 (38.657%)	765 (39.474%)		
Male	0	3,785 (61.088%)	2,612 (61.343%)	1,173 (60.526%)		
Age, y	0	57.836 ± 12.501	57.823 ± 12.480	57.864 ± 12.550	−0.119	0.905
BMI, kg/m^2^ (mean, sd)	1 (0.02%)	24.742 ± 3.632	24.735 ± 3.606	24.756 ± 3.690	−0.209	0.834
Smoke					13.185	<0.001
0	0	4,460 (71.982%)	3,125 (73.391%)	1,335 (68.885%)		
1	0	1736 (28.018%)	1,133 (26.609%)	603 (31.115%)		
Diabetes					0.006	0.939
0	0	5,104 (82.376%)	3,506 (82.339%)	1,598 (82.456%)		
1	0	1,092 (17.624%)	752 (17.661%)	340 (17.544%)		
Hypertension					2.548	0.11
0	0	5,192 (83.796%)	3,590 (84.312%)	1,602 (82.663%)		
1	0	1,004 (16.204%)	668 (15.688%)	336 (17.337%)		
Infarction					0	1
0	0	5,622 (90.736%)	3,864 (90.747%)	1758 (90.712%)		
1	0	574 (9.264%)	394 (9.253%)	180 (9.288%)		
Ventilation (hours)	0	13.000 [7.000, 18.000]	12.000 [7.000, 17.875]	14.000 [8.000, 19.000]		<0.001
ICU stay (hours)	21 (0.3%)	70.000 [48.000, 96.000]	68.000 [48.000, 96.000]	72.000 [59.000, 112.000]		<0.001
Postoperative hospital stay (days)	0	8.000 [7.000, 11.000]	8.000 [7.000, 10.000]	9.000 [7.000, 12.000]		<0.001
CPB					34.348	<0.001
0	0	3,109 (50.178%)	2,244 (52.701%)	865 (44.634%)		
1	0	3,087 (49.822%)	2014 (47.299%)	1,073 (55.366%)		
LCOS					7.914	0.005
0	0	5,663 (91.398%)	3,921 (92.085%)	1742 (89.886%)		
1	0	533 (8.602%)	337 (7.915%)	196 (10.114%)		
Season					56.188	<0.001
Autumn	0	1,505 (24.290%)	1,084 (25.458%)	421 (21.723%)		
Spring	0	1749 (28.228%)	1,237 (29.051%)	512 (26.419%)		
Summer	0	1,575 (25.420%)	1,110 (26.069%)	465 (23.994%)		
Winter	0	1,367 (22.063%)	827 (19.422%)	540 (27.864%)		
AQI	0	99.271 ± 53.237	94.118 ± 49.014	110.592 ± 59.985	−10.588	<0.001
PM_2.5_	0	59.680 ± 44.242	54.969 ± 39.553	70.030 ± 51.632	−11.408	<0.001
PM_10_	0	120.342 ± 78.222	113.327 ± 74.264	135.754 ± 84.277	−10.07	<0.001
O_3_	0	75.916 ± 45.379	78.194 ± 45.338	70.912 ± 45.079	5.884	<0.001
SO_2_	0	25.219 ± 28.335	22.397 ± 25.387	31.417 ± 33.098	−10.655	<0.001
CO	0	0.979 ± 0.525	0.928 ± 0.482	1.091 ± 0.595	−10.576	<0.001
T_max_	0	19.988 ± 10.945	20.344 ± 10.851	19.204 ± 11.113	3.77	<0.001
T_min_	0	10.173 ± 10.686	10.857 ± 10.464	8.671 ± 11.014	7.354	<0.001
Humidity	0	53.807 ± 18.865	53.714 ± 18.928	54.012 ± 18.730	−0.578	0.563
Quality					146.956	<0.001
1	0	551 (8.893%)	423 (9.934%)	128 (6.605%)		
2	0	3,293 (53.147%)	2,384 (55.989%)	909 (46.904%)		
3	0	1,612 (26.017%)	1,065 (25.012%)	547 (28.225%)		
4	0	430 (6.940%)	243 (5.707%)	187 (9.649%)		
5	0	235 (3.793%)	105 (2.466%)	130 (6.708%)		
6	0	75 (1.210%)	38 (0.892%)	37 (1.909%)		

HAP, hospital-acquired pneumonia; ICU, Intensive care unit; BMI, Body mass index; CPB, cardiopulmonary bypass; LCOS, Low cardiac output syndrome; PM_2.5_, Particulate matter with diameter ≤ 2.5 μm; PM_10_, Particulate matter with diameter ≤ 10 μm; SO_2_, Sulfur dioxide; O_3_, Ozone; CO, carbon monoxide; T_max_, Daily maximum temperature of day; T_min,_ Daily minimum temperature of day. Data are mean ±SD, median (IQR) or *n*/*N* (%).

### The distributed lag non-linear model

Because HAP was a binary patient-level outcome, we fitted a logistic distributed lag non-linear model (DLNM) using a generalized linear model with a binomial distribution and a logit link function. Let ( $Y_{i}$ ) denote the HAP status of patient (*i*). The model was specified as follows:

$$Y_{i}∼\text{Bernoulli}(p_{i})$$

$$\begin{array}{l} \text{logit}(p_{i})=\log(\frac{p_{i}}{1-p_{i}})=\beta_{0}+{\mathrm{CB}}_{i}({\mathrm{PM}}_{2.5})\\ +\beta_{1}{\mathrm{Age}}_{i}+\beta_{2}{\mathrm{Sex}}_{i}+\beta_{3}{\mathrm{BMI}}_{i}+ \end{array}$$

$$\begin{array}{l} \mathrm{ns}({\mathrm{CO}}_{i},3)+\mathrm{ns}({\mathrm{SO}}_{2i},3)+\mathrm{ns}(O_{3i},3)\\ +\mathrm{ns}({\text{Humidity}}_{i},3)+\mathrm{ns}({\text{Temperature}}_{i},3) \end{array}$$

Where $p_{i}=P(Y_{i}=1)$ is the probability of HAP for patient *i*, and $CB_{i}(PM_{2.5})$ represents the cross-basis function for PM_2.5_ exposure over the perioperative exposure window. The PM_2.5_ cross-basis was constructed using natural cubic splines in both the exposure-response and lag-response dimensions. In the xposure-response dimension, internal knots were placed at the 30th and 60th percentiles of the PM_2.5_ distribution. In the lag-response dimension, a natural cubic spline with 3 degrees of freedom was used. The perioperative PM_2.5_ exposure window included six exposure days: 2 days after surgery, 1 day after surgery, the day of surgery, and 1–3 days before surgery.

All covariates were entered simultaneously into a single multivariable logistic DLNM. Covariates were selected *a priori* based on clinical relevance, previous evidence, and their potential roles as confounders or prognostic factors. Patient-level variables, including age, sex, and BMI, were adjusted to account for baseline susceptibility to HAP and differences in perioperative risk profiles. Co-pollutants and meteorological variables, including CO, SO₂, O₃, relative humidity, and temperature, were included because they may be associated with PM_2.5_ exposure and may also independently affect respiratory outcomes. These environmental variables were modeled using natural cubic splines with 3 degrees of freedom to allow for potential non-linear relationships.

Because the model used a logit link function, the associations between PM_2.5_ exposure and HAP were reported as odds ratios (ORs) with 95% confidence intervals (CIs).

### Statistical analysis

Because this retrospective cohort study was based on existing clinical records, no prospective sample-size calculation was performed. We instead assessed the adequacy of the available sample size using the commonly applied events-per-parameter principle for multivariable regression modeling. The primary logistic DLNM included 28 estimated model parameters after accounting for the PM_2.5_ cross-basis function and spline terms for co-pollutants and meteorological variables. Based on a conventional target of 10–20 outcome events per estimated parameter, approximately 280–560 HAP events would be required for stable model estimation. Historical hospitalization records from 2011–2012 suggested an HAP incidence of approximately 10–20%; therefore, a sample size of approximately 2,800–5,600 patients was considered adequate. To maximize precision, all eligible patients during the study period were included, and 6,196 patients were included in the primary complete-case logistic DLNM analysis.

For statistical assessment, categorical variables were summarized as counts and percentages and compared between patients with and without HAP using χ^2^ tests or Fisher’s exact tests, as appropriate. Continuous variables were summarized as means with standard deviations (SDs) or medians with interquartile ranges (IQRs), according to their distributions. Between-group comparisons for continuous variables were performed using Student’s t-tests for normally distributed variables and Wilcoxon rank-sum tests for non-normally distributed variables. Correlations among daily air pollutants and meteorological variables were evaluated using Spearman’s rank correlation coefficients.

Because the respiratory effects of short-term air pollution may be delayed and non-linear, we used a logistic distributed lag non-linear model (DLNM) to estimate the association between perioperative PM_2.5_ exposure and HAP risk. HAP was modeled as a binary patient-level outcome using a generalized linear model with a binomial distribution and a logit link function, and effect estimates were reported as odds ratios (ORs) with 95% confidence intervals (CIs).

Subgroup analyses were conducted to examine whether the association between perioperative PM_2.5_ exposure and HAP differed across clinically relevant patient characteristics and perioperative factors. The primary logistic DLNM was refitted within strata defined by age (≤45, 46–60, and >60 years), BMI (≤23.9 and >23.9 kg/m^2^), sex, season of surgery, history of infarction, cardiopulmonary bypass, smoking status, diabetes, and hypertension. Seasons were defined according to calendar months as spring (March–May), summer (June–August), autumn (September–November), and winter (December–February). Subgroup-specific ORs and 95% CIs were estimated using the same PM_2.5_ cross-basis specification and covariate adjustment strategy as in the primary model, except that the stratification variable was not additionally adjusted for within the corresponding subgroup model. Differences between subgroup-specific estimates were assessed using Z tests based on the estimated log(ORs) and their standard errors. R (version 4.2.2) was employed for all statistical assessments, and the significance threshold was set as two-tailed *p < 0.05*.

### Sensitivity analyses

Sensitivity analyses were conducted across six analytical domains to evaluate the robustness and interpretability of the primary findings.

1. Co-pollutant adjustment. The influence of co-pollutant adjustment was assessed by fitting single-, two-, and three-pollutant models with different combinations of CO, SO₂, and O₃ in addition to the PM_2.5_ cross-basis term.
2. Exposure-range robustness. Low- and high-exposure models were constructed to assess whether the exposure–lag–response pattern was consistent across different PM_2.5_ exposure ranges, using 50 μg/m^3^ as the primary cutoff.
3. Expanded clinical covariate adjustment. An expanded covariate-adjusted sensitivity model was fitted by additionally including smoking status, diabetes, hypertension, cardiopulmonary bypass, and duration of mechanical ventilation on top of the covariates included in the primary model. Perioperative antibiotic management followed a stepwise institutional strategy, starting with lower-level agents and escalating according to clinical symptoms, laboratory indicators, and imaging findings when clinically indicated. The ICU nurse-to-patient ratio followed a fixed institutional staffing standard. Therefore, these care-related factors were not modeled as additional individual-level covariates.
4. DLNM specification and lag-window robustness. The robustness of the DLNM specification was examined using three complementary analyses. First, we varied the PM_2.5_ cross-basis parameters to assess sensitivity to the choice of lag–response degrees of freedom and exposure–response knot locations. Alternative models used lag–response degrees of freedom of 2 and 4 with the primary exposure knots, and exposure knots at the 25th and 75th percentiles or at the 25th, 50th, and 75th percentiles with the primary lag–response degrees of freedom. Second, the PM_2.5_ exposure window was extended from lag 0–5 to lag 0–7 to evaluate whether a longer perioperative exposure window materially changed the exposure–lag–response pattern. Third, an auxiliary linear-trend analysis was performed by specifying the exposure–response dimension as linear while keeping the lag–response structure and covariate adjustment strategy unchanged. This analysis was used only to assess monotonic trends across increasing PM_2.5_ levels and did not replace the primary non-linear DLNM analysis.
5. Temporal, seasonal, and holiday-related robustness. Temporal robustness was evaluated using three additional analyses. First, calendar time was additionally adjusted to account for potential long-term temporal trends in pollution levels, surgical practice, hospital admission patterns, and perioperative care. Second, season was additionally adjusted to account for broad seasonal variation in air pollution, meteorological conditions, respiratory vulnerability, and clinical practice. Seasons were defined according to calendar months as spring (March–May), summer (June–August), autumn (September–November), and winter (December–February). Third, observations during major Chinese public holiday periods, including the Spring Festival, Labor Day, and National Day holidays, were excluded to evaluate whether holiday-related irregularities in pollution levels, hospital admission, surgical scheduling, staffing, or care patterns influenced the observed association. Potential seasonal effect modification was further assessed using a likelihood ratio test comparing models with and without the PM_2.5_ cross-basis-by-season interaction term.
6. Pandemic-period robustness. Pandemic-period sensitivity analyses were conducted to address potential changes in hospital practice, infection-control measures, ICU management, surgical scheduling, and patient mix during the COVID-19 pandemic. The study period was classified according to the date of surgery into a pre-pandemic period (2014–2019) and a pandemic period (2020–2023), and the primary logistic DLNM was refitted separately within each period using the same PM_2.5_ cross-basis specification and covariate adjustment strategy as in the primary analysis. Patient-level active COVID-19 infection status was not used for stratification because patients with active COVID-19 infection were not scheduled for elective surgery in this setting.

### Model diagnostics

Model diagnostics were conducted to assess the stability and transparency of the DLNM analyses. For the multi-pollutant DLNM models, we reported the number of observations, number of model parameters, residual degrees of freedom, AIC, BIC, and convergence status. Because the primary DLNM used unpenalized natural cubic splines in a standard logistic regression framework, model complexity was summarized using the number of model parameters and residual degrees of freedom rather than penalized effective degrees of freedom.

To evaluate potential multicollinearity in the multi-pollutant models, variance inflation factors were calculated for the raw pollutant and meteorological variables included in each model. Because PM_2.5_ was modeled using a cross-basis function, PM_2.5_ was represented by the mean concentration across the perioperative exposure window for the VIF calculation. In addition, condition indices were calculated using the full model matrix for each DLNM.

## Results

Table 1 summarizes various clinical and demographic parameters of the 6,196 adult patients. In total, 1938 (31.28%) patients were diagnosed with HAP. Among the total study population, 1736 (28.018%) had a history of smoking, 1,092 (17.624%) of diabetes, 1,004 (16.204%) of hypertension, and 574 (9.264%) of infarction. The median ventilation duration was 13 h (6, 17), and ICU stay was 70 h [48, 96].

Pollutant data (PM_2.5_, SO_2,_ and O_3_) were obtained from Jinan city from 2014 to 2023 (Figure 2). It was observed that the average levels of O_3_, PM_10_, CO, PM_2.5_, and SO_2_ were 75, 120, 0.979, 59, and 25 μg/m^3^, respectively. The daily T_max_, relative humidity, and T_min_ were 19.988 °C, 53.807%, and 10.173 °C, respectively. However, whether PM_2.5_ levels improved annually remains unclear; PM_2.5_ in 2014 was substantially worse than in 2023 (Figure 2). During the study, the daily PM_2.5_ levels were more than the current and next NAAQS target of 75 μg/m^3^ on 915 days (35%) and 50 μg/m^3^ on 1,581 days (60%), respectively. There were 812 days (31%) where PM_10_ values were > 150 μg/m3. The Spearman correlation indicated a substantial link between daily PM_2.5_, PM_10_, SO_2_, and O_3_ (Figure 3).

**Figure 2:**
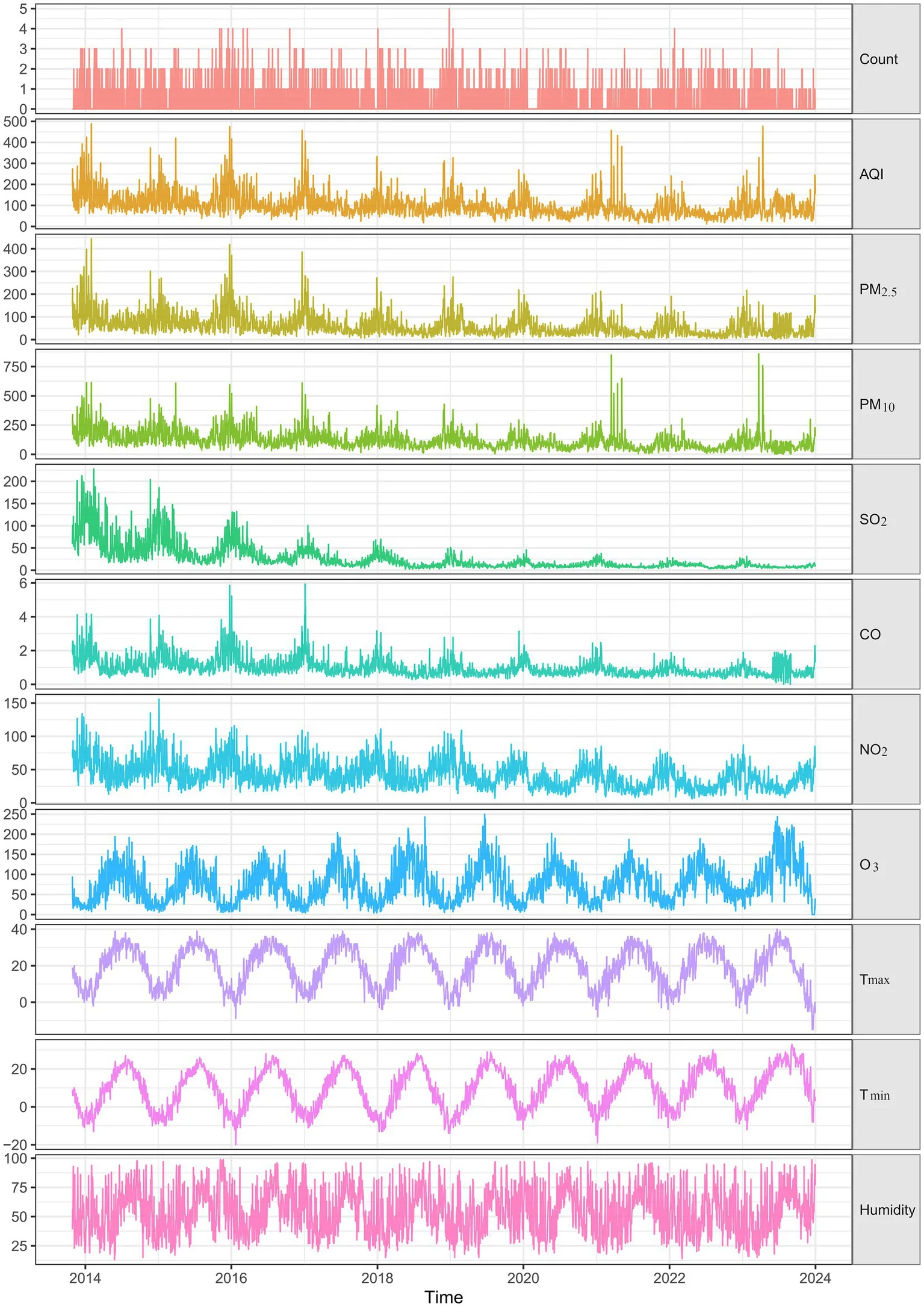
The air quality in Jinan city from 2014 to 2023. Count: daily number of surgeries, PM_2.5_: Particulate matter with diameter ≤ 2.5 μm, PM_10_, Particulate matter with diameter ≤ 10 μm; SO_2_, Sulfur dioxide; O_3_, Ozone; CO, carbon monoxide; T_max_, Daily maximum temperature of day; T_min_, Daily minimum temperature of day.

**Figure 3:**
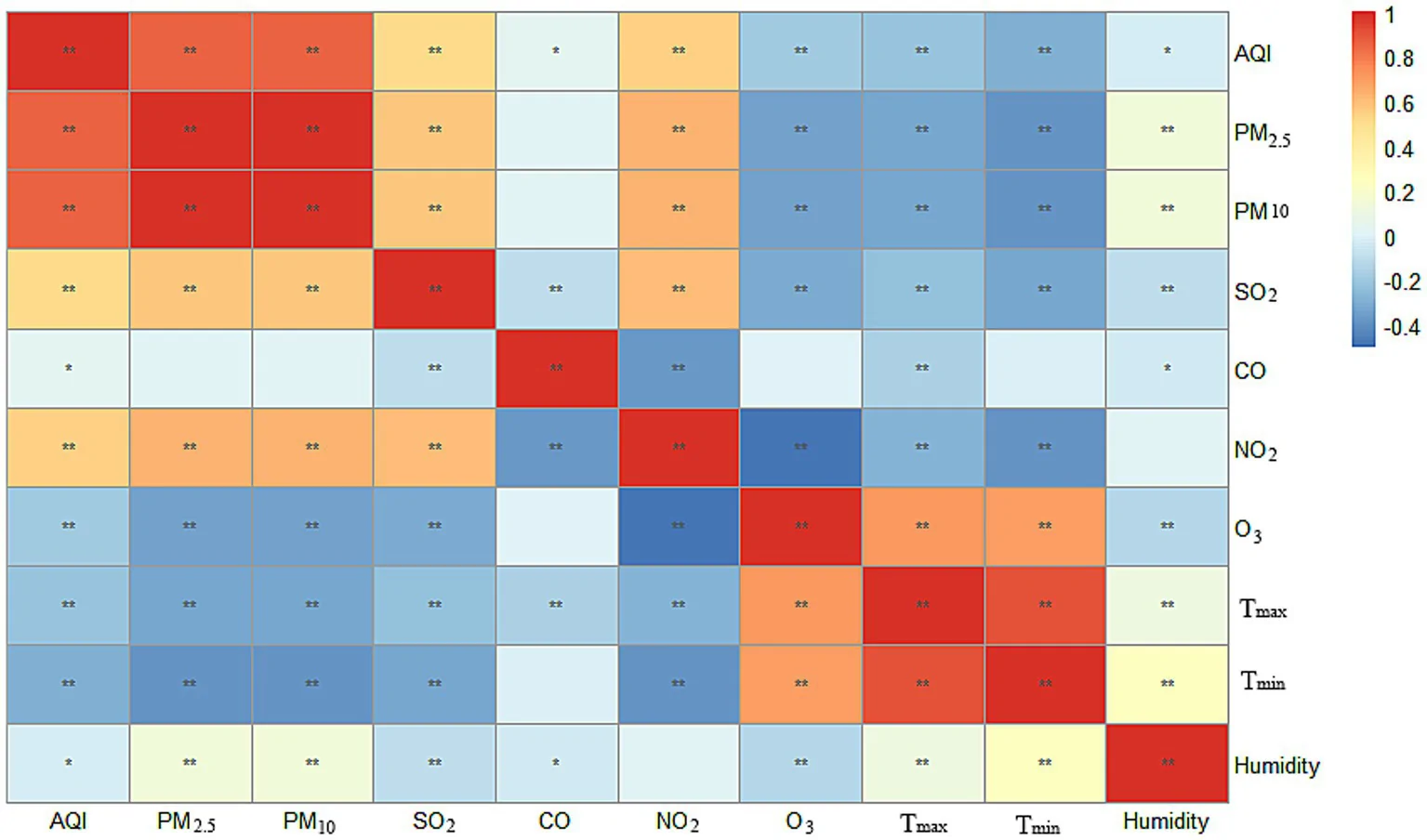
Spearman’s correlation coefficients between daily air pollutants and weather conditions in Jinan, 2014–2023. PM_2.5_, Particulate matter with diameter ≤ 2.5 μm; PM_10_, Particulate matter with diameter ≤ 10 μm; SO_2_, Sulfur dioxide; O_3_, Ozone; CO, carbon monoxide; T_max_, Daily maximum temperature of day; T_min_, Daily minimum temperature of day. *<0.05; **<0.01.

### Main findings

To assess how pollutants affect HAP onset, the impact of PM_2.5_ was modelled in DLNMs. It was found that PM_2.5_ had an acute effect on HAP, with the most significant effect observed at lag 1 day within the perioperative exposure window (Table 2; Figure 4). At a PM2.5 concentration of 20 μg/m^3^ compared with the reference concentration of 10 μg/m^3^, the OR was 1.116 (95% CI: 0.986–1.262) on lag 0 day and 1.076 (95% CI: 1.012–1.144) on lag 1 day, with the effect dissipating by lag 2 day (OR: 1.040; 95% CI: 0.974–1.111). At PM2.5 concentrations of 50 μg/m^3^ or higher, the effect became stronger and persisted for a longer duration. The OR value was 1.226 (95% CI: 0.938–1.604) on lag 0-day, peaked at 1.216 (95% CI: 1.065–1.387) on lag 1-day, and remained at 1.187 (95% CI: 1.031–1.368) on lag 2-day, dissipating by lag 3-day (OR: 1.127; 95% CI: 0.977–1.299). A dose–response association was observed between PM_2.5_ content and HAP. The OR increases with increasing PM_2.5_ concentration over the range 0–100 μg/m^3^. For concentrations above 100 μg/m^3^, the growth trend gradually slows down (Figure 5B). Moreover, the 1-day to 2-day lag period corresponds to the length of stay in the ICU, as HAP is generally diagnosed 2 days after surgery. A cumulative effect of PM_2.5_ was observed on HAP. At a PM2.5 concentration of 30 μg/m^3^ compared with the reference concentration of 10 μg/m^3^, the OR increased from 1.205 (95% CI: 0.972–1.492) on lag 0-day to a maximum of 1.542 on lag 0–3 days (95% CI: 1.086–2.191), thereafter decreasing to 1.446 (95% CI: 0.969–2.159) on lag 0–5 days. At a PM2.5 concentration of 50 μg/m^3^ compared with the reference concentration of 10 μg/m^3^, the OR was 1.226 (95% CI: 0.938–1.604) on lag 0-day, peaking at 2.071 on lag 0–4 days (95% CI: 1.323–3.24), and thereafter declining to 1.953 (95% CI: 1.2–3.179) on lag 0–5 days (Figure 5A).

**Table 2:** Odds ratios of HAP at different PM_2.5_ concentrations compared with 10 μg/m^3^ across lag times.

PM_2.5_ (ug/m^3^)	lag0	lag1	lag2	lag3	lag4	lag5
10		Reference	Reference	Reference	Reference	Reference
20	1.116 [0.986, 1.262]	1.076 [1.012, 1.144]	1.040 [0.974, 1.111]	1.011 [0.946, 1.081]	0.989 [0.938, 1.043]	0.969 [0.874, 1.075]
30	1.205 [0.972, 1.492]	1.142 [1.026, 1.270]	1.085 [0.967, 1.216]	1.034 [0.921, 1.160]	0.989 [0.902, 1.084]	0.948 [0.792, 1.135]
40	1.239 [0.957, 1.606]	1.188 [1.045, 1.351]	1.134 [0.988, 1.302]	1.073 [0.934, 1.233]	1.007 [0.901, 1.124]	0.940 [0.757, 1.168]
50	1.226 [0.938, 1.604]	1.216 [1.065, 1.387]	1.187 [1.031, 1.368]	1.127 [0.977, 1.299]	1.039 [0.928, 1.163]	0.943 [0.755, 1.179]
60	1.195 [0.916, 1.560]	1.234 [1.083, 1.406]	1.241 [1.081, 1.426]	1.186 [1.031, 1.364]	1.076 [0.964, 1.201]	0.951 [0.765, 1.183]
70	1.165 [0.892, 1.522]	1.251 [1.099, 1.424]	1.294 [1.129, 1.484]	1.245 [1.084, 1.430]	1.114 [1.000, 1.241]	0.960 [0.774, 1.191]
80	1.138 [0.869, 1.491]	1.266 [1.112, 1.442]	1.344 [1.173, 1.541]	1.302 [1.134, 1.495]	1.150 [1.033, 1.280]	0.970 [0.782, 1.202]

ORs were estimated using the logistic distributed lag non-linear model. PM_2.5_ exposure was modeled using a cross-basis function over the perioperative exposure window. Data are presented as ORs with 95% confidence intervals. The reference concentration was 10 μg/m^3^.

**Figure 4:**
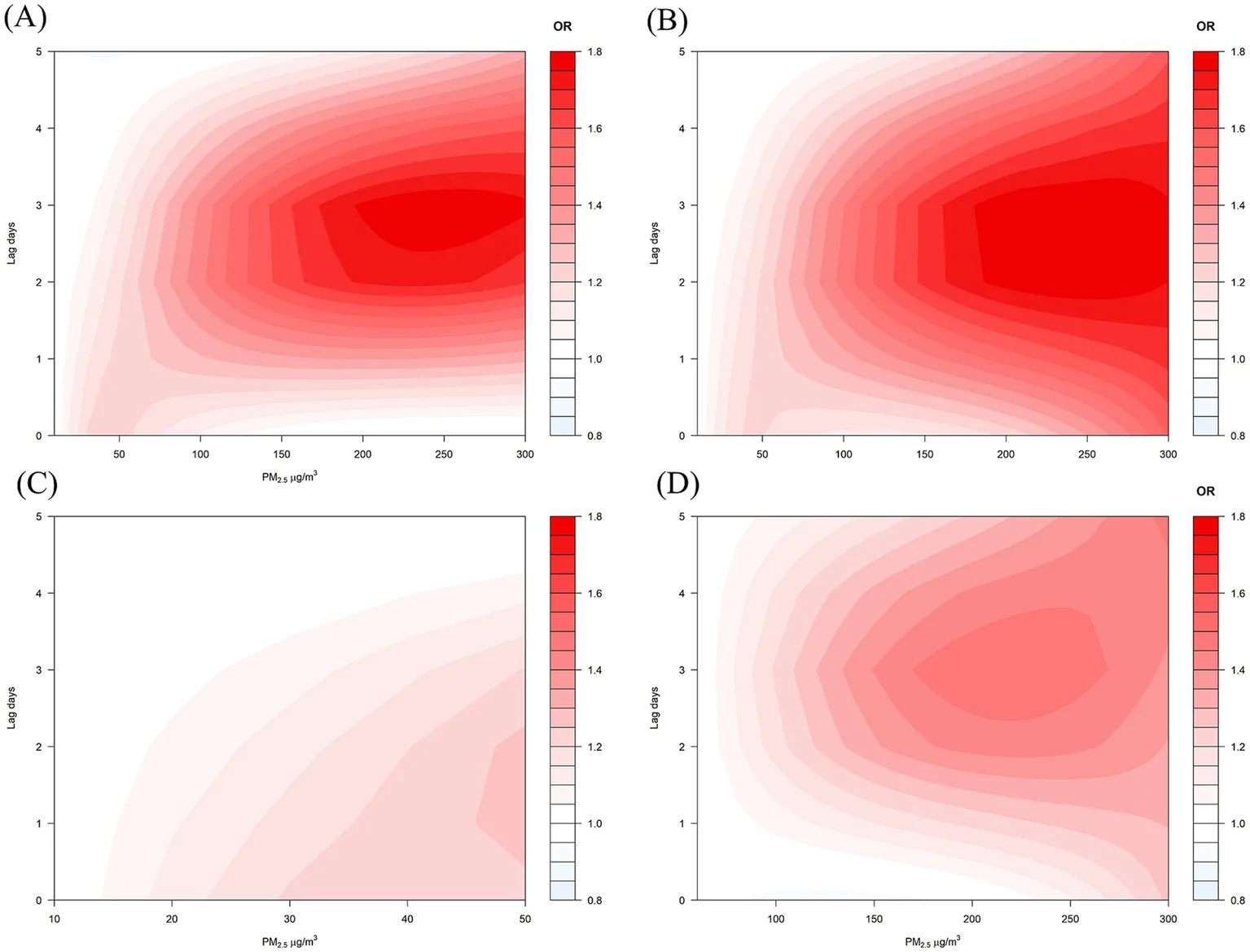
Contour plots showing the adjusted effect of different models. All panels use the same OR color scale ranging from 0.8 to 1.8. Contour plots showing the adjusted effect of main model **(A)**, single-pollutant of PM_2.5_ **(B)**, low-exposure model **(C)** and high-exposure model **(D)** on the OR of HAP on different lag days. OR, odds ratio; HAP, Hospital-acquired pneumonia, PM_2.5_, Particulate matter with diameter ≤ 2.5 μm.

**Figure 5:**
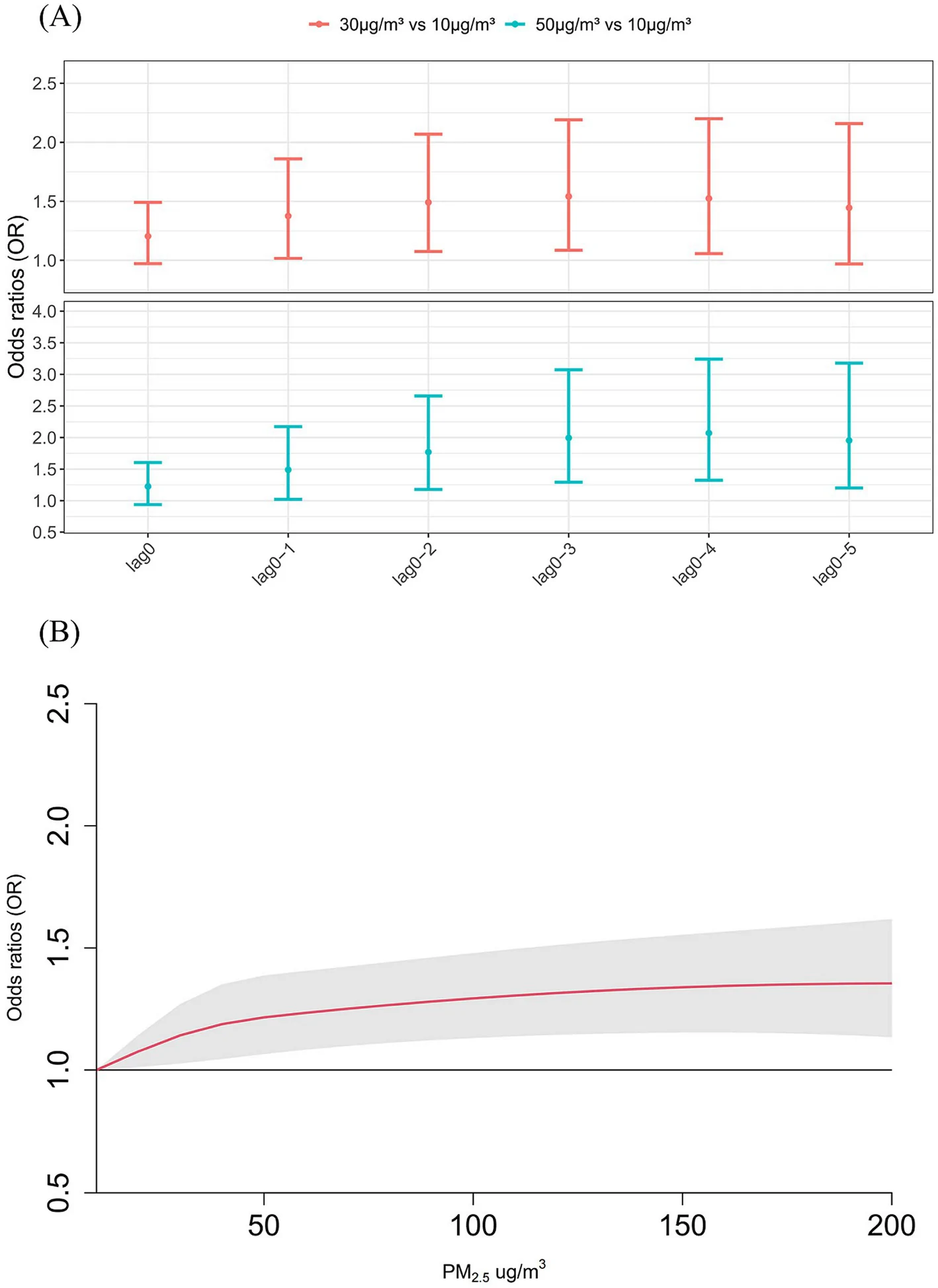
Estimated exposure–response curves for short-term exposures to PM_2.5_. **(A)** Cumulative-response curve showing the cumulative effect of PM_2.5_ concentrations of 30 μg/m^3^ and 50 μg/m^3^ compared with the reference concentration of 10 μg/m^3^ on the OR for HAP across increasing lag days. **(B)** Exposure-response curve showing increase in the OR for HAP with increasing PM_2.5_ concentration on lag-1 day. Gray area indicates 95% CIs. PM_2.5_: Fine particulate matter; HAP: hospital-acquired pneumonia.

### Sensitivity analyses

Sensitivity analyses across the six analytical domains generally supported the robustness of the primary findings.

In the co-pollutant sensitivity analyses, the association between perioperative PM_2.5_ exposure and HAP remained generally consistent across PM_2.5_-only, two-pollutant, and three-pollutant models (Table 3). At lag day 1 and a PM_2.5_ level of 30 μg/m^3^, the PM_2.5_-only model showed an increased risk of HAP (OR = 1.138, 95% CI: 1.038–1.248). After additional adjustment for individual co-pollutants, the estimated associations remained positive, with ORs of 1.165 (95% CI: 1.052–1.291) in the PM_2.5_ + CO model, 1.107 (95% CI: 1.004–1.221) in the PM_2.5_ + SO₂ model, and 1.136 (95% CI: 1.035–1.247) in the PM_2.5_ + O₃ model. Three-pollutant models also showed generally consistent positive associations between PM_2.5_ and HAP.

**Table 3:** Odds ratios of HAP with exposure to different combinations of pollutants.

Model		Estimate	SE	95% CI	OR
Main analysis^a^		0.133	0.054	[1.026, 1.270]	1.142
Single-pollutant analysis^b^		0.129	0.047	[1.038, 1.248]	1.138
Two-pollutant model^c^	PM_2.5_ + SO_2_	0.102	0.050	[1.004, 1.221]	1.107
	PM_2.5_ + CO	0.153	0.052	[1.052, 1.291]	1.165
	PM_2.5_ + O_3_	0.128	0.048	[1.035, 1.247]	1.136
Three-pollutant model	PM_2.5_ + SO_2_ + CO	0.129	0.054	[1.024, 1.264]	1.138
	PM_2.5_ + SO_2_ + O_3_	0.100	0.051	[1.001, 1.221]	1.106
	PM_2.5_ + CO + O_3_	0.155	0.053	[1.053, 1.296]	1.168

For this analysis, lag time was fixed at lag 1 day and the PM_2.5_ concentration was set at 30 μg/m^3^. SO_2_: Sulfur dioxide, O_3_: Ozone, CO: carbon monoxide. ^a^The main analysis modeled the effects of all four pollutants, including PM_2.5_, SO_2_, CO and O_3._^b^The single-pollutant analysis modeled the effect of PM_2.5_ alone ^c^The two-pollutant analysis modeled the effect of PM_2.5_ with SO_2_, CO, or O_3._

In the exposure-range robustness analysis using 50 μg/m^3^ as the cutoff, the exposure–lag–response pattern was generally consistent across the low- and high-exposure models. The low-exposure model showed a higher OR than the high-exposure model, suggesting that the observed association was not driven solely by extreme PM_2.5_ concentrations.

After expanded clinical and perioperative covariate adjustment, including smoking status, diabetes, hypertension, cardiopulmonary bypass, and duration of mechanical ventilation, the overall exposure–lag–response pattern was not materially changed, although the effect estimates were slightly attenuated. The elevated-risk region remained mainly concentrated at higher PM_2.5_ concentrations and lag days 2–4 (Supplementary Figure 1).

The findings were also robust to alternative DLNM specifications and lag-window definitions. When alternative lag–response degrees of freedom and alternative exposure–response knot placements were used, the smoothness and magnitude of the risk surface varied slightly, but the elevated-risk region remained mainly concentrated at higher PM_2.5_ concentrations and delayed lag days, particularly around lag days 2–4 (Supplementary Figure 2). When the exposure window was extended from lag 0–5 to lag 0–7, the elevated-risk region remained mainly concentrated at higher PM_2.5_ concentrations and lag days 2–4, and no additional elevated-risk region was observed at the newly extended lag days (Supplementary Figure 3 and Supplementary Table 1). In the auxiliary linear-trend analysis, increasing PM_2.5_ levels were associated with higher HAP risk mainly at delayed lag days, with significant positive trends at lag days 1–4 and the strongest and most consistent trends at lag days 2–4. This pattern was consistent with the primary non-linear DLNM results (Supplementary Figure 4 and Supplementary Table 2).

Temporal, seasonal, and holiday-related sensitivity analyses showed that the association was not materially altered after additional adjustment for calendar time or season (Supplementary Figures 5A,B). After excluding patients who underwent surgery during major Chinese public holiday periods, including the Spring Festival, Labor Day, and National Day holidays, 41 patients were removed and 6,150 patients remained; the overall exposure–lag–response pattern was nearly unchanged (Supplementary Figure 5C). The formal PM_2.5_-by-season interaction test did not provide evidence that the PM_2.5_–HAP association differed by season (P for interaction = 0.650).

In the pandemic-period sensitivity analysis, the overall exposure–lag–response patterns were broadly consistent between the pre-pandemic period (2014–2019) and the pandemic period (2020–2023). In the pre-pandemic period, elevated risks were mainly observed at higher PM_2.5_ concentrations and delayed lag days, particularly around lag days 2–3, which was consistent with the primary analysis. In the pandemic period, positive associations were also observed, although the elevated-risk region appeared to be more evident around lag day 1 and the estimates were less precise (Supplementary Figure 6).

### Subgroup analyses

The positive relationship between PM_2.5_ and HAP was stronger in patients aged ≥ 60 years (OR: 1.196, 95% CI: 1.02–1.403), whereas there was no association with age ≤ 45 or 46–60 years. Moreover, the history of diabetes was significantly related to HAP (OR: 1.35, 95% CI: 1.031–1.766). For BMI > 23.9 kg/m^2^, PM_2.5_ also afects HAP significantly (OR: 1.183, 95% CI: 1.029–1.361). The gender, season, or CPB indicated no modifying effect between PM_2.5_ and HAP (Figure 6).

**Figure 6:**
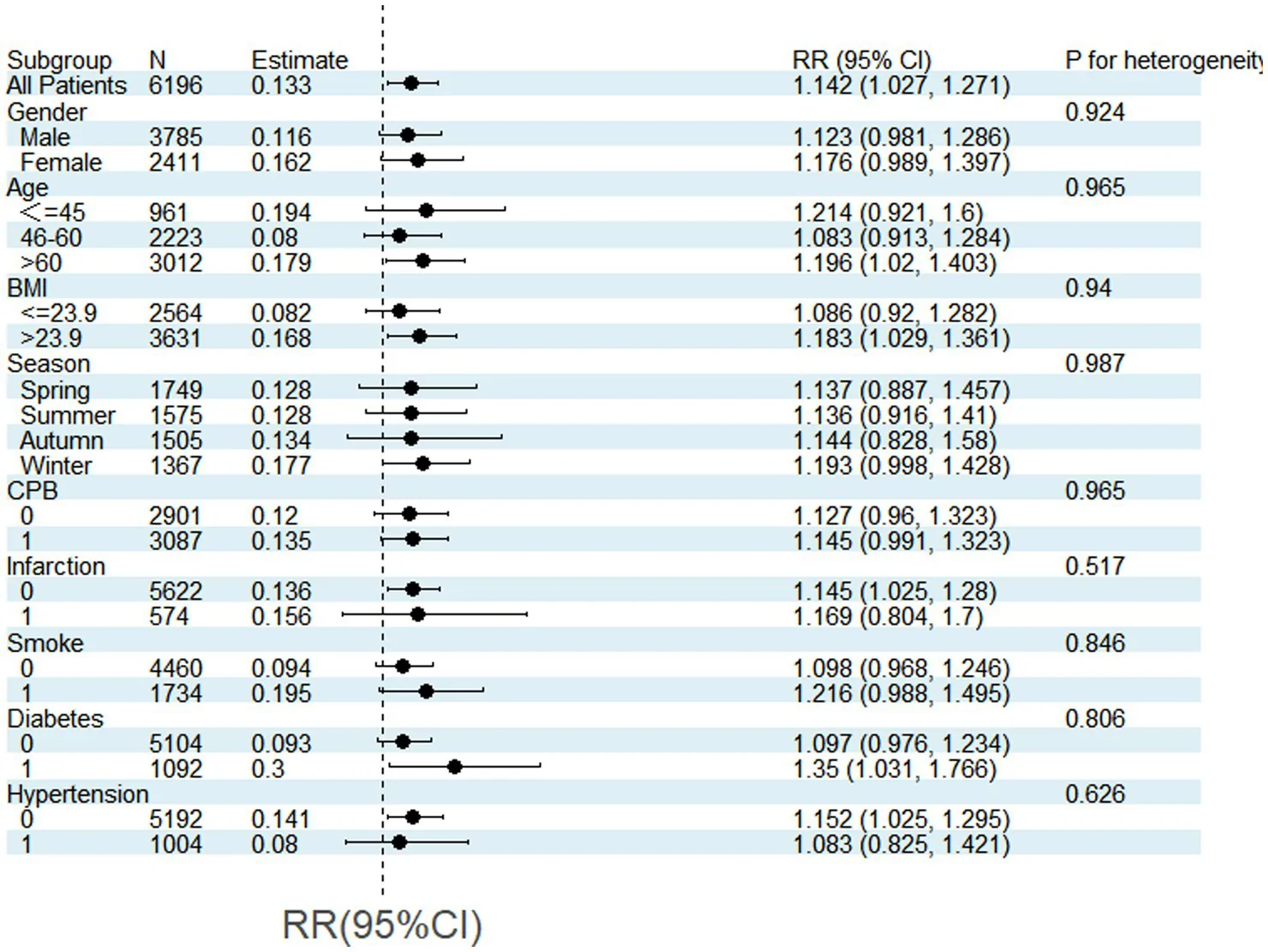
Subgroup-specific odds ratios for HAP at a PM2.5 concentration of 30 μg/m^3^ compared with the reference concentration of 10 μg/m^3^. HAP, hospital-acquired pneumonia, ICU, Intensive care unit, BMI, Body mass index, CPB, cardiopulmonary bypass, LCOS, Low cardiac output syndrome.

### Model diagnostics

Collinearity and model diagnostics did not indicate severe multicollinearity or numerical instability in the primary and multi-pollutant DLNM models. The maximum VIFs for the raw pollutant and meteorological variables ranged from 1.60 to 3.40, and the maximum condition indices of the full model matrices ranged from 7.25 to 13.79. All models converged successfully (Supplementary Table 3).

## Discussion

Previous literature has indicated an association between short-term exposure to pollutants and the prognosis in hospitalized patients; however, the impact of pollutants on specific patients remains elusive. This is the first study to report a significant and delayed acute effect of PM_2.5_ on adult HAP patients undergoing cardiac surgery (n = 6,196). Furthermore, it was observed that a PM2.5 concentration of 30 μg/m^3^ compared with the reference concentration of 10 μg/m^3^ was associated with 14.2% higher odds of HAP. The influence was delayed, with the strongest effect observed at lag 1 day within the perioperative exposure window. These data support the hypothesis that improving ICU air quality may reduce the onset of HAP.

There are strong links between HAP, ambient pollution, and cardio-respiratory decline in patients, nascent associations with pollution, and shared biological pathways (28, 29). It has been observed that air pollutants can stimulate systemic or local inflammation (30). Furthermore, oxidative stress mediators can induce a local inflammatory response, either being directly stimulated by particulate compounds or indirectly by activated cellular enzymes. The influence of pollution on ICU patients has not been comprehensively studied; therefore, this study focuses on vulnerable patients undergoing cardiac surgery for more than 2 days in the ICU post surgery. Several studies have indicated pneumonia onset over the first few days following exposure to PM_2.5_, with risk returning to baseline within a week (31, 32). This finding is consistent with the results of the present study; however, the strongest effect was observed at lag 1 day within the perioperative exposure window, unlike our previous study, which found the largest lag effect 2 or 3 days before VAP onset.

Various lag structures (i.e., 1–5 days for PM_2.5_) associated with distinct PM_2.5_ levels (10–80 μg/m^3^) for health outcome assessments require further research. Based on previous studies, the delays for PM_2.5_ or O_3_ remain plausible (33, 34). The reduced lag for PM may be corroborated by an experimental investigation indicating a slight increase of 5% in the older adults population within 24 to 48 h following exposure to concentrated ambient air particles (19). However, the association between PM_2.5_ exposure and HAP remained evident after co-pollutant adjustment. The main effect estimate for HAP was a 14.2% (95% CI: 2.6–27%) higher odds at a PM2.5 concentration of 30 μg/m^3^ compared with the reference concentration of 10 μg/m^3^ on lag 1 day, exceeding those found in general population studies, potentially attributable to the high-risk group analyzed. In China, the current NAAQS for daily PM_2.5_ is 75 μg/m^3^. Jinan is an industrial city predominantly centered on manufacturing, and its traditional practice of burning coal during winter, both of which put it among the top 10 most polluted Chinese cities. In 2014, the mean PM_2.5_ concentration was 90 μg/m^3^, which progressively decreased to 54 μg/m^3^ before 2024. However, this value still exceeds the WHO intermediate target level 3 of 37.5 μg/m^3^. A study indicated that PM_2.5_ content > 15 μg/m^3^ can enhance the mortality rate (35). Meanwhile, other heavily polluted cities also showed enhanced risk for respiratory diseases (36, 37). Therefore, the presented study may provide benchmarks for heavily polluted cities.

This epidemiological study also found that several patient features were linked with a stronger association between PM2.5 and HAP, with older patients showing a higher effect (38). Elderly patients with cardiac disorders who are hospitalized long-term frequently endure multiple concurrent chronic conditions, including stroke, cancer, respiratory diseases, hypertension, and diabetes mellitus. Research indicates that mortality from influenza and respiratory disorders progresses with advancing age, peaking in individuals over 65 years old (39, 40). Furthermore, it has been observed that older adults individuals with preexisting respiratory conditions are more vulnerable to pneumonia, as the combined impact of several chronic diseases substantially impacts an individual’s activities, quality of life, and mortality risk in this demographic (41). Moreover, the patterns in the burden of HAP varied across age groups. In individuals aged > 65, fragility was more common, and ICU prognosis was worse (42). This outcome resembled findings from another study, which indicated that ambient PM pollution is a significant determinant in increasing lower respiratory infections (LRI) and mortality, particularly in females aged ≥ 70; ambient PM emerged as the primary risk factor, accounting for 11.7% of LRI-related deaths (43).

In the present subgroup analysis, sex did not show a significant modifying effect on the association between PM_2.5_ exposure and HAP, which is consistent with previous evidence suggesting that the cumulative lag effects of PM_2.5_ may be observed in both men and women (44). Smoking, however, may exacerbate lung inflammation and increase susceptibility to respiratory injury. According to a UK Biobank cohort study, smoking exposure reduced lung function, thus validating the strong link between smoking and COPD (45). Moreover, smoking behaviors are PM_2.5_ mediators_,_ leading to COPD (46).

Diabetes was another susceptibility-related factor in the present study. In patients with type 2 diabetes mellitus (T2DM), short-term AP exposure significantly aggravated HAP incidence. The mechanisms include systemic inflammation, beta-cell dysfunction, hepatic insulin resistance, glucose metabolism, oxidative stress, and alterations in insulin signaling. The air contaminants mostly deposit inside the alveoli and bronchioles of the respiratory system. These particles can induce cytokine and inflammatory mediator secretion, causing inflammation and increasing blood C-reactive protein (CRP) levels, aggravating lung inflammation (47). Foreign agents modulate macrophages to secrete cytokines, which promote localized inflammation in the lung’s alveolar cells because of polluted PM exposure. This localized inflammation has the potential to develop into systemic inflammation (48).

Similarly, air pollutants like NO_2_, PM_2.5_, and O_3_ have been linked with increased adiposity (49), which is consistent with the present study, indicating that obesity can enhance the link between AP and inflammation. Obesity is a proinflammatory condition, and being overweight or obese may increase susceptibility to an exaggerated oxidative stress and inflammatory response. The presence of AP and obesity results in a multiplicative impact. Further, many hormones, including adiponectin, leptin, and resistin, which have an inverse correlation with obesity, may exacerbate the negative effects of AP (50). Alterations in the respiratory system of obese individuals may also explain the increased susceptibility to AP. The literature and a weight loss trial have demonstrated a correlation between obesity and increased tidal volume, resulting in higher exposure to inhaled particles in individuals with obesity (51).

This study has numerous significant strengths. (1) The findings corroborate the link between short-term PM_2.5_ exposure and HAP in regions with varying degrees of AP. (2) A larger sample size and data covering a decade provide more objective and precise conclusions. (3) Compared to other studies on healthy participants, this investigation included postoperative patients with comprehensive data. In the DLNM model, the potential confounders were adjusted, and bias was reduced. To our knowledge, this is the first epidemiology study to examine the correlation between short-term PM_2.5_ exposure and HAP in ICU patients undergoing cardiac surgery.

However, these findings also have some limitations. First, this was an observational, single-center study, and the results require replication and validation through additional multi-center epidemiological investigations. In addition, the diagnosis of HAP was contingent upon ICU clinical physicians, potentially resulting in a delay relative to the actual onset of HAP. Second, selection bias related to cohort construction cannot be fully ruled out. Patients with inadequate ICU observation windows, preoperative pneumonia, or unavailable exposure assignment were excluded to ensure reliable HAP ascertainment and valid PM_2.5_ exposure assessment. However, these exclusions may have resulted in an analytic cohort that differed from excluded patients in clinical severity, perioperative course, or exposure characteristics. Because complete and comparable information was not available for all excluded patients, we were unable to directly compare included and excluded patients or quantify the direction and magnitude of this potential bias.

Exposure misclassification was unavoidable because all exposure data were derived from fixed-site monitors rather than personal measurements. However, this type of exposure measurement error is typically nondifferential in longitudinal studies and may tend to underestimate the effects of air pollutants.

In addition, the use of city-wide average PM_2.5_ concentrations could not capture intra-urban spatial heterogeneity in air pollution exposure. Previous studies have shown that PM_2.5_ concentrations may vary within urban areas and that using fixed-site monitors or city-level average concentrations as surrogates for individual exposure may introduce exposure misclassification (52, 53). Because detailed geocoded residential addresses and district-specific exposure estimates were not available, spatially varying exposure–response analyses could not be performed in the present study.

Several temporal, hospital-operation, and care-pressure factors could not be fully captured. Although we performed sensitivity analyses adjusting for calendar time or season, excluding major Chinese public holiday periods, and stratifying analyses by pandemic period, local influenza or respiratory virus surveillance data and direct time-varying measures of hospital workload were not available. In our hospital, perioperative management, infection-control procedures, HAP diagnosis and reporting, and medical and nursing staff allocation followed standardized institutional protocols throughout the study period. However, daily hospital occupancy, ICU bed pressure, actual nurse-to-patient ratio, and staffing pressure were unavailable. Therefore, residual confounding related to unmeasured respiratory epidemic activity, hospital workload, or care-pressure indicators cannot be completely excluded.

Duration of mechanical ventilation was not included as a core covariate in the primary model because it may reflect postoperative clinical severity, early respiratory deterioration, or clinical response after surgery rather than a conventional baseline confounder. However, it was included in the expanded clinical covariate-adjusted sensitivity analysis, and the overall exposure–lag–response pattern was not materially changed.

## Conclusion

In summary, this study found that short-term exposure to PM_2.5_ enhances HAP risk among ICU-hospitalized adults. Furthermore, PM_2.5_ exhibited cumulative and delayed impact, indicating that enhancements in air quality before and during ICU admission may affect respiratory infections. Moreover, this correlation was more pronounced among the older, patients with higher BMI, and individuals with diabetes. Therefore, PM_2.5_ poses a risk even at concentrations well below current national air quality standards, suggesting that air quality in the ICU may need reevaluation for vulnerable groups.

## Data Availability

The raw data supporting the conclusions of this article will be made available by the authors, without undue reservation.

## Glossary

AAP: Ambient air pollution
HAP: hospital-acquired pneumonia
ICU: intensive care unit
AQI: Air Quality Index
PM_2.5_: particulate matter 2.5
PM_10_particulate matter 10
SO_2_: sulfur dioxide
NO_2_: nitrogen dioxide
CO: carbon monoxide
O_3_: ozone
NAAQS: National Ambient Air Quality Standard
AP: Air pollution
COPD: chronic obstructive pulmonary disease
IQR: inter-quartile range
SD: standard deviation
OR: odds ratio
DLNM: the distributed lag non-linear model
BMI: body mass index
CPB: cardiopulmonary bypass
LRI: lower respiratory infections
T2DM: type 2 diabetes
CRP: C-reactive protein
