# Supplementary material for: Association between short-term fine particulate matter exposure and hospital-acquired pneumonia risk in critically ill post-cardiac surgery patients: a 10-year cohort study using distributed lag non-linear models
Source: Front Public Health. 2026 Oct 22;14:1867760. doi: 10.3389/fpubh.2026.1867760 (PMC13736763; doi:10.3389/fpubh.2026.1867760)
Supplement: Supplementary file 1 [file Data_Sheet_1.DOCX]

Supplementary Materials

Contents

[Supplementary Figure 1. Sensitivity analysis with expanded covariate adjustment.	2](#_Toc32410)

[Supplementary Figure 2. Sensitivity analysis using alternative PM₂.₅ cross-basis specifications.	3](#_Toc6138)

[Supplementary Figure 3. Sensitivity analysis using an extended lag window from lag 0 to lag 7.	4](#_Toc31262)

[Supplementary Table 1. Lag-specific odds ratios from the extended lag-window DLNM	5](#_Toc14740)

[Supplementary Figure 4. Exposure–lag–response association from the auxiliary linear-exposure logistic DLNM.	6](#_Toc14202)

[Supplementary Table 2. Lag-specific odds ratios and P values for linear trend from the auxiliary linear-exposure logistic DLNM.	7](#_Toc32545)

[Supplementary Figure 5. Sensitivity analysis adjusting for temporal factors and excluding major public holidays.	9](#_Toc16787)

[Supplementary Table 3. Collinearity and model diagnostics for the primary and multi-pollutant DLNM models	11](#_Toc21948)

# Supplementary Figure 1. Sensitivity analysis with expanded covariate adjustment.

**Supplementary Figure 1. Sensitivity analysis with expanded covariate adjustment.**

**Note: The x-axis represents PM_2.5_ concentration, the y-axis represents DLNM lag days, and the color scale represents the odds ratio (OR) for HAP relative to the reference PM_2.5_ concentration.**

# **Supplementary Figure 2. Sensitivity analysis using alternative PM_2.5_ cross-basis specifications.**

**Supplementary Figure 2. Sensitivity analysis using alternative PM_2.5_ cross-basis specifications.**

**Note:** Exposure–lag–response surfaces were estimated using alternative specifications of the PM_2.5_ cross-basis function. The four panels represent: (A) a natural cubic spline with 2 degrees of freedom for the lag-response dimension and exposure-response knots at the 30th and 60th percentiles; (B) a natural cubic spline with 3 degrees of freedom for the lag-response dimension and exposure-response knots at the 25th and 75th percentiles; (C) a natural cubic spline with 3 degrees of freedom for the lag-response dimension and exposure-response knots at the 25th, 50th, and 75th percentiles; and (D) a natural cubic spline with 4 degrees of freedom for the lag-response dimension and exposure-response knots at the 30th and 60th percentiles.

# **Supplementary Figure 3. Sensitivity analysis using an extended lag window from lag 0 to lag 7.**

**Supplementary Figure 3. Sensitivity analysis using an extended lag window from lag 0 to lag 7.**

**Note: The x-axis represents PM_2.5_ concentration, the y-axis represents DLNM lag days, and the color scale represents the odds ratio (OR) for HAP relative to the reference PM_2.5_ concentration.**

# **Supplementary Table 1. Lag-specific odds ratios from the extended lag-window DLNM**

****Supplementary Table 1. Lag-specific odds ratios from the extended lag-window DLNM****

| PM_2.5_ (ug/m3) | lag0 | lag1 | lag2 | lag3 | lag4 | lag5 | lag6 | lag7 |
| --- | --- | --- | --- | --- | --- | --- | --- | --- |
| 10 | 1.000 [1.000, 1.000] | 1.000 [1.000, 1.000] | 1.000 [1.000, 1.000] | 1.000 [1.000, 1.000] | 1.000 [1.000, 1.000] | 1.000 [1.000, 1.000] | 1.000 [1.000, 1.000] | 1.000 [1.000, 1.000] |
| 20 | 1.119 [1.007, 1.244] | 1.081 [1.016, 1.150] | 1.047 [1.000, 1.095] | 1.017 [0.963, 1.073] | 0.993 [0.940, 1.048] | 0.974 [0.932, 1.019] | 0.960 [0.911, 1.012] | 0.947 [0.866, 1.036] |
| 30 | 1.217 [1.013, 1.462] | 1.152 [1.034, 1.283] | 1.092 [1.010, 1.182] | 1.041 [0.947, 1.143] | 0.997 [0.907, 1.097] | 0.962 [0.891, 1.039] | 0.932 [0.851, 1.021] | 0.905 [0.775, 1.058] |
| 40 | 1.269 [1.016, 1.584] | 1.200 [1.054, 1.366] | 1.136 [1.033, 1.248] | 1.076 [0.961, 1.204] | 1.020 [0.910, 1.144] | 0.969 [0.884, 1.063] | 0.921 [0.826, 1.027] | 0.876 [0.727, 1.057] |
| 50 | 1.278 [1.015, 1.607] | 1.227 [1.074, 1.403] | 1.176 [1.068, 1.295] | 1.120 [0.998, 1.257] | 1.058 [0.941, 1.190] | 0.991 [0.902, 1.089] | 0.923 [0.826, 1.032] | 0.858 [0.708, 1.040] |
| 60 | 1.268 [1.010, 1.592] | 1.245 [1.091, 1.420] | 1.214 [1.105, 1.333] | 1.168 [1.044, 1.307] | 1.102 [0.983, 1.236] | 1.019 [0.930, 1.118] | 0.931 [0.835, 1.037] | 0.844 [0.699, 1.019] |
| 70 | 1.258 [1.002, 1.579] | 1.261 [1.106, 1.437] | 1.250 [1.140, 1.371] | 1.215 [1.087, 1.357] | 1.145 [1.023, 1.283] | 1.047 [0.957, 1.146] | 0.938 [0.844, 1.044] | 0.832 [0.691, 1.002] |
| 80 | 1.248 [0.992, 1.570] | 1.275 [1.118, 1.455] | 1.285 [1.173, 1.408] | 1.260 [1.128, 1.407] | 1.187 [1.060, 1.329] | 1.074 [0.982, 1.175] | 0.946 [0.851, 1.051] | 0.822 [0.683, 0.989] |

Note: Data are presented as ORs with 95% confidence intervals. The reference concentration was 10 μg/m³.

# **Supplementary Figure 4. Exposure–lag–response association from the auxiliary linear-exposure logistic DLNM.**

****Supplementary Figure 4. Exposure–lag–response association from the auxiliary linear-exposure logistic DLNM.****

**Note: The x-axis represents PM_2.5_ concentration, the y-axis represents DLNM lag days, and the color scale represents the odds ratio (OR) for HAP relative to the reference PM_2.5_ concentration.** All panels use the same OR color scale ranging from 0.8 to 1.8.

# **Supplementary Table 2. Lag-specific odds ratios and P values for linear trend from the auxiliary linear-exposure logistic DLNM.**

**Supplementary Table 2. Lag-specific odds ratios and P values for linear trend from the auxiliary linear-exposure logistic DLNM.**

| PM_2.5_ (ug/m3) | lag0 | lag1 | lag2 | lag3 | lag4 | lag5 |
| --- | --- | --- | --- | --- | --- | --- |
| 10 | 1.000 [1.000, 1.000] | 1.000 [1.000, 1.000] | 1.000 [1.000, 1.000] | 1.000 [1.000, 1.000] | 1.000 [1.000, 1.000] | 1.000 [1.000, 1.000] |
| 20 | 0.994 [0.976, 1.012] | 1.012 [1.004, 1.021] | 1.026 [1.018, 1.034] | 1.030 [1.021, 1.038] | 1.023 [1.017, 1.029] | 1.011 [0.999, 1.023] |
| 30 | 0.988 [0.953, 1.025] | 1.025 [1.008, 1.042] | 1.053 [1.037, 1.069] | 1.060 [1.043, 1.077] | 1.046 [1.034, 1.059] | 1.022 [0.998, 1.047] |
| 40 | 0.982 [0.930, 1.037] | 1.038 [1.012, 1.064] | 1.080 [1.056, 1.105] | 1.091 [1.066, 1.117] | 1.070 [1.051, 1.089] | 1.033 [0.997, 1.071] |
| 50 | 0.976 [0.907, 1.050] | 1.051 [1.016, 1.086] | 1.108 [1.075, 1.142] | 1.123 [1.088, 1.160] | 1.094 [1.069, 1.121] | 1.045 [0.996, 1.096] |
| 60 | 0.970 [0.886, 1.063] | 1.064 [1.020, 1.109] | 1.137 [1.095, 1.181] | 1.157 [1.112, 1.203] | 1.119 [1.087, 1.153] | 1.056 [0.995, 1.121] |
| 70 | 0.964 [0.864, 1.076] | 1.077 [1.024, 1.132] | 1.167 [1.115, 1.220] | 1.191 [1.135, 1.249] | 1.145 [1.105, 1.186] | 1.068 [0.995, 1.147] |
| 80 | 0.958 [0.844, 1.089] | 1.090 [1.029, 1.156] | 1.197 [1.136, 1.262] | 1.226 [1.160, 1.296] | 1.171 [1.124, 1.220] | 1.080 [0.994, 1.174] |
| P for linear trend | 0.514 | 0.004 | <0.001 | <0.001 | <0.001 | 0.07 |

Note: Data are presented as ORs with 95% confidence intervals. The reference concentration was 10 μg/m³.

**To evaluate the trend across increasing PM_2.5_ levels, we performed an auxiliary linear-trend analysis using a logistic distributed lag model. In this auxiliary model, the exposure-response dimension of the PM_2.5_ cross-basis was specified as linear using `argvar = list(fun = "lin")`, while the lag-response dimension was modeled using the same natural cubic spline with 3 degrees of freedom as in the primary model. The covariate adjustment strategy was also kept the same as in the primary model, including age, sex, BMI, CO, SO_2_, O_3_, relative humidity, and temperature. This auxiliary model was used only to evaluate the linear trend across increasing PM_2.5_ levels and did not replace the primary non-linear DLNM estimates reported in Table 2.**

**The auxiliary linear-exposure DLNM showed a delayed positive trend between increasing PM_2.5_ levels and HAP risk. No significant linear trend was observed at lag day 0 (P for linear trend = 0.514). A significant positive trend was observed at lag day 1 (P = 0.004), and stronger positive trends were observed at lag days 2–4 (all P < 0.001). The trend at lag day 5 did not reach statistical significance (P = 0.070). The corresponding exposure–lag–response surface is shown in Supplementary Figure 4. These findings were consistent with the primary non-linear DLNM results, suggesting that the association between higher perioperative PM_2.5_ exposure and HAP risk was mainly delayed and concentrated at lag days 2–4.**

# **Supplementary Figure 5. Sensitivity analysis adjusting for temporal factors and excluding major public holidays.**

**Supplementary Figure 5. Sensitivity analysis adjusting for temporal factors and excluding major public holidays.**

**Note:** **Exposure–lag–response associations between perioperative PM_2.5_ exposure and HAP risk were estimated using three temporal sensitivity models. The same cross-basis specification and covariate adjustment strategy as in the primary analysis were used in both periods. The three panels represent: (A) a model additionally adjusted for calendar time using a natural cubic spline; (B) a model additionally adjusted for season; and (C) a model excluding patients who underwent surgery during major Chinese public holidays, including the Spring Festival, Labor Day, and National Day holidays. The x-axis represents PM_2.5_ concentration, the y-axis represents DLNM lag days, and the color scale represents the odds ratio (OR) for HAP relative to the reference PM_2.5_ concentration.** All panels use the same OR color scale ranging from 0.8 to 1.8.

# **Supplementary Figure 6. Pandemic-period stratified exposure–lag–response association between perioperative PM_2.5_ exposure and HAP.**

**Supplementary Figure 6. Pandemic-period stratified exposure–lag–response association between perioperative PM_2.5_ exposure and HAP.**

**Note: The primary logistic DLNM was refitted separately for the pandemic period (A: 2020–2023) and the pre-pandemic period (B: 2013–2019). The x-axis represents PM_2.5_ concentration, the y-axis represents DLNM lag days, and the color scale represents the odds ratio (OR) for HAP relative to the reference PM_2.5_ concentration. The same cross-basis specification and covariate adjustment strategy as in the primary analysis were used in both periods.**

# **Supplementary Table 3. Collinearity and model diagnostics for the primary and multi-pollutant DLNM models**

**Supplementary Table 3. Collinearity and model diagnostics for the primary and multi-pollutant DLNM models**

| Model | Number_of_parameters | Residual_df | AIC | BIC | Maximum_VIF_raw_variables | Maximum_condition_index_raw_variables | Maximum_condition_index_full_model_matrix |
| --- | --- | --- | --- | --- | --- | --- | --- |
| Main model: PM_2.5_ + CO + SO_2_ + O_3_ + humidity + temperature | 28 | 6167 | 7456.20 | 7644.69 | 3.40 | 4.37 | 13.79 |
| Only PM_2.5_ model: PM_2.5_ + humidity + temperature | 19 | 6176 | 7461.80 | 7589.70 | 1.60 | 2.04 | 7.25 |
| PM_2.5_ + CO | 22 | 6173 | 7462.33 | 7610.43 | 2.28 | 2.71 | 12.30 |
| PM_2.5_ + SO_2_ | 22 | 6173 | 7456.26 | 7604.35 | 2.07 | 2.63 | 8.02 |
| PM_2.5_ + O_3_ | 22 | 6173 | 7459.31 | 7607.40 | 3.40 | 3.56 | 7.82 |
| PM_2.5_ + O_3_ + CO | 25 | 6170 | 7459.48 | 7627.77 | 3.40 | 3.97 | 12.90 |
| PM_2.5_ + O_3_ + SO_2_ | 25 | 6170 | 7455.76 | 7624.04 | 3.40 | 3.97 | 8.55 |
| PM_2.5_ + CO + SO_2_ | 25 | 6170 | 7456.90 | 7625.19 | 2.55 | 3.20 | 13.32 |

SO_2_: Sulfur dioxide, O_3_: Ozone, CO: carbon monoxide.
